# Intraspecific Genetic Diversity Analyses of Yam (*Dioscorea polystachya* Turcz.) Based on DUS Traits and SSR Molecular Markers

**DOI:** 10.1002/ece3.72295

**Published:** 2025-10-16

**Authors:** Jin Gao, Jiangli Zhang, Jiage Wang, Yingying Chang, Zhao Qin, Lintao Sun, Mingjun Li, Qingxiang Yang

**Affiliations:** ^1^ College of Life Sciences Henan Normal University Xinxiang China; ^2^ Henan International Joint Laboratory of Agricultural Microbial Ecology and Technology Henan Normal University Xinxiang China; ^3^ Engineering Laboratory of Green Medicinal Material Biotechnology of Henan Province Henan Normal University Xinxiang China

**Keywords:** *Dioscorea polystachya* Turcz., DUS, genetic diversity, genetic structure, germplasm identification, SSR

## Abstract

Yam ( *Dioscorea polystachya* Turcz.) is an asexually reproduced food and traditional Chinese medicinal crop with extensive genomic variability. However, the detailed characterization of genetic diversity among different yam germplasm samples is still insufficient. This study evaluated the genetic divergence and genetic structure of 113 *D. polystachya* accessions collected from 17 provinces in China based on 50 distinctness, uniformity, and stability (DUS) traits and 19 simple sequence repeat (SSR) markers. All the selected varieties were categorized into three groups based on morphological characteristics and further validated by principal component analysis. Furthermore, 14 core traits, including 6 leaf traits, 4 tuber traits, 3 bulbil traits, and 1 stem trait, were selected to increase field inspection efficiency. SSR fingerprinting, utilizing 19 highly polymorphic markers, successfully distinguished all 113 yam varieties, revealing relatively high levels of genetic variation. Interestingly, the optimal genetic structure defined three groups, whereas a finer‐scale model consistently classified the varieties into five groups, corroborating the genotypic cluster analysis. Furthermore, this study preliminarily identified 10 groups of potential heterotypic synonyms and 13 groups of potential homonyms among the yam accessions. These results demonstrate that the 19 selected SSR markers, in conjunction with DUS traits, can effectively discriminate the 113 *D. polystachya* varieties. Our findings provide critical insights for the conservation of pure breeds and the utilization of *Dioscorea* germplasm resources.

## Graphical abstract

The 113 *D. polystachya* accessions exhibited substantial genetic diversity, clearly distinguished using 19 SSR markers and 50 DUS traits. A refined set of 14 core DUS traits was established, significantly improving variety identification efficiency. These selected markers and traits provide valuable tools for genetic research and support the utilization of germplasm in future breeding programs.

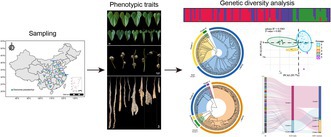

## 1 Introduction

Yam (*Dioscorea* spp.) has been extensively cultivated in East Asia for both food and medicinal purposes, with its tubers being rich in starch, mucin, and medicinal compounds (Lebot et al. 2019; Sautour et al. 2007). As the fourth most important tuber crop, following potatoes, cassava, and sweet potatoes, it has exceptionally high economic value (Shewry 2003). In the Chinese Pharmacopeia, the authentic herb “yam” is defined exclusively as *Dioscorea polystachya* Turcz. (Wang, Gu, et al. 2022), a temperate‐adapted species primarily cultivated in China and Japan. Despite its mode of asexual propagation, yam exhibits remarkable biodiversity, environmental adaptability, and phenotypic plasticity (Epping and Laibach 2020), a result of ancient agricultural practices in which wild tubers underwent sustained vegetative propagation and selection pressure. These processes resulted in morphological and biochemical alterations, particularly in tuber characteristics, that ultimately led to the development of distinct varieties (Wu et al. 2014). The rich yam germplasm reserves present expanded allelic variation and new possibilities for genetic exploration and cultivar development. In China, locally adapted varieties of *D. polystachya* across various regions exhibit abundant phenotypic and genetic diversity, often resulting in mixed‐variety cultivation practices. Yam yield and quality are inherently linked to genotype, encompassing genetic purity, morphological uniformity, germination potential, and physiological vigor. However, mixed and adulterated yam tubers pose significant challenges for farmers and subsequent market distribution. It is increasingly imperative to establish a comprehensive framework for differentiating *D. polystachya* cultivars, which exhibit high abundance yet minimal distinguishing characteristics. Such a framework is essential to establish effective and accurate identification protocols that maintain purebred traits and support the documentation of germplasm resources.

Traditionally, the authentication of *Dioscorea* species has relied primarily on their morphological traits, which are significantly influenced by environmental factors and local cultivation settings. The traits typically employed for yam variety identification include leaf morphology, bulbil traits, tuber attributes, disease resistance, and other distinguishing features (Cao et al. 2021). The International Union for the Protection of New Varieties of Plants (UPOV) requires DUS (Distinctness, Uniformity, Stability) testing as an essential prerequisite for varietal registration and intellectual property protection (Yang et al. 2021). China has developed and introduced the first domestic standard for DUS testing of yams, titled the Guidelines for Conducting Tests on Distinctness, Uniformity, and Stability in Yam ( *Dioscorea alata* L.; *Dioscorea polystachya* Turcz.; *Dioscorea japonica* Thunb.; NY/T 2495‐2013). The existing DUS assessment system encounters significant challenges, requiring substantial time, financial, and labor investments while being vulnerable to environmental and methodological variables that affect field‐based morphological evaluations (Jamali et al. 2019; Zhang, Yang, et al. 2022).

DNA fingerprinting tests have demonstrated efficacy in genotype identification and authentication due to their high accuracy and efficacy. Diverse molecular markers, including simple sequence repeat (SSR), inter simple sequence repeat (ISSR), sequence‐related amplified polymorphism (SRAP), random amplified polymorphic DNA (RAPD), amplified fragment length polymorphism (AFLP), and single nucleotide polymorphism (SNP) markers, have been developed for the identification of *Dioscorea* species (Arnau et al. 2017; Cao et al. 2021; Mignouna et al. 2005; Wu et al. 2014; Wang, Wang, et al. 2022; Feng et al. 2024). Among the different molecular markers, SSR markers are widely utilized in ecology, biology, and genetics due to their codominant inheritance pattern and ubiquitous genomic distribution in distinguishing genome loci and multiple alleles (Otoo et al. 2015). Recent research on the origins and genetics of yam suggests that *D. polystachya* Turcz. probably originated in China, where it is the most prevalent species with the largest cultivated area (Cao et al. 2021). Currently, several studies have investigated the genetic diversity of *D. alata* and *D. polystachya* Turcz. (Arnau et al. 2017; Massawe and Temu 2023; Wu et al. 2019; Feng et al. 2024). However, there remains a scarcity of research on genetic variation in *D. polystachya* Turcz. using integrated molecular and morphological markers across a broad spectrum of germplasm resources. Significant interspecific genetic differences have been observed among different species within the genus *Dioscorea*, leading to clustering results that often follow the species classification patterns. However, compared with the substantial genomic divergence among *Dioscorea* species, intraspecific variation within *D. polystachya* varieties is relatively minor. As *D. polystachya* is one of China's most extensively cultivated yam species, it is imperative to elucidate its genetic structure and diversity to enhance our understanding of its genetic traits and environmental adaptability, thus offering robust scientific guidance for its agricultural production. At the same time, the consistency between molecular‐based genotypic profiling and field DUS testing in yam germplasm has received limited attention, making it urgently necessary to establish integrated molecular platforms for variety authenticity and purity identification and germplasm genetic analysis.

*Dioscorea polystachya* germplasm resources exhibit significant variability at the phenotypic, biochemical, and DNA levels, indicating substantial genetic diversity. The aims of this study were (i) to assess the morphological variability of *D. polystachya* accessions in China and (ii) to develop an SSR fingerprinting method utilizing 19 SSRs to distinguish and authenticate 113 *D. polystachya* candidate varieties. Simultaneously, DUS testing was conducted using either 50 DUS traits or 14 core traits to evaluate the precision and effectiveness of SSR fingerprinting. Selecting a core set of DUS traits can substantially reduce labor expenses and increase the efficiency of differentiating various *D. polystachya* accessions. Molecular‐based SSR testing can further enhance the detection effectiveness of DUS testing methods while reducing costs, thereby addressing a critical gap in plant cultivar authentication.

## 2 Materials and Methods

### 2.1 *Dioscorea polystachya* Accessions and DUS Traits Assessment

**FIGURE 1:**
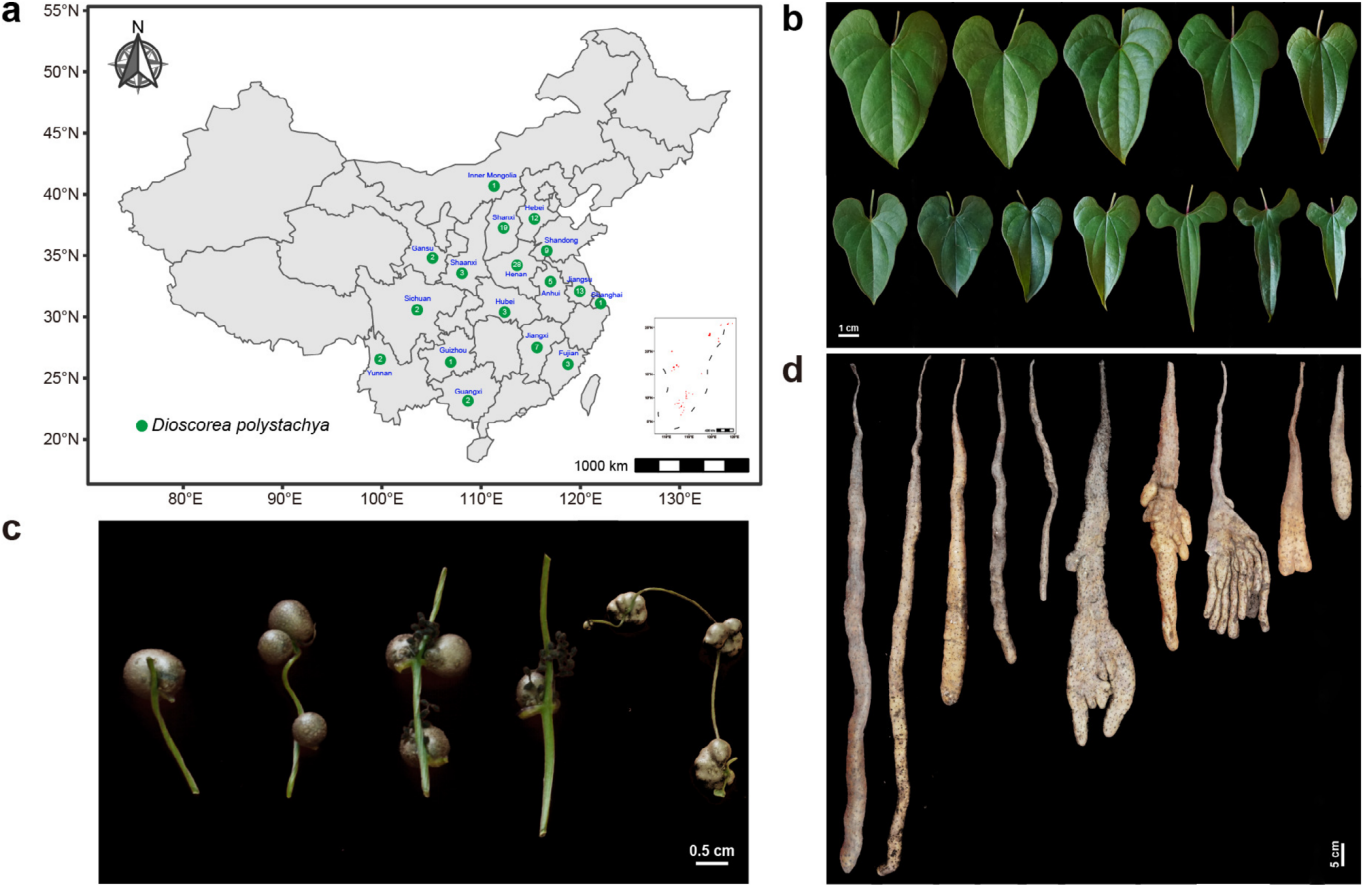
Geographical distribution and phenotypic diversity of yam germplasm resources in China. (a) Geographic origin and number of accessions collected per province, numbers indicate the total accessions collected in each province; (b–d) representative morphological variation in leaves (b), bulbils (c), and tubers (d).

A total of 113 yam accessions maintained and propagated at the Wenxian Institute of Agricultural Sciences were utilized to analyze accession structure and genetic diversity. Yam accession details, including names and geographical origins, were provided (Table S1, Figure 1a). The accessions were planted in April 2022 and subsequently harvested in November at the Wenxian germplasm resource base (112°58′57″ E, 34°56′55″ N) located in Jiaozuo, Henan Province, China. The experimental planting was set up in ridges, with 20 cm separating each individual plant and a 1.2 m distance between ridges. Disease‐ and pest‐free tuber segments (60–100 g) were used as “propagules.” Each yam accession was assessed in five replicates with six individual plants per replicate, and the standard horticultural practices were performed regularly to ensure optimal plant growth conditions. A total of 50 DUS phenotypic traits were recorded across yam leaves, stems, flowers, petioles, bulbils, tubers, and disease resistance, comprising 35 qualitative and 15 quantitative traits. Based on NY/T 2495‐2013 DUS guidelines for yam, qualitative traits were classified into distinct grades according to their descriptive states, with each grade assigned a specific score (Table 1). For quantitative traits, grading intervals for 15 characteristics were established using the least significant difference (LSD) method. The mean value of each trait served as the midpoint of the central grade. Intervals were extended symmetrically with a class width greater than twice LSD_0.05_ and then adjusted empirically to establish final grade ranges. The frequency and proportion of each grade were subsequently calculated. All collected phenotypic data and the assessment scores are listed in Table 1. Phenotypic analyses were conducted on 30 plants per yam accession, with their average value recorded for subsequent evaluation.

**TABLE 1:** Detailed information of 50 DUS testing traits in 113 yam accessions identification.

Traits	Classes (codes)	Max	Min	Range	Mean	Standard deviation (SD)	The average coefficient of variation (CV%)	Shannon–Weaver diversity index
**T1: Leaf density**	3 = Sparse, 5 = Medium, 7 = Dense	7	5	2	6.84	0.54	7.92	0.32
T2: Branch number	3 = Minority, 5 = Medium, 7 = Majority	7	5	2	6.82	0.57	8.33	0.35
T3: Stem spirality	1 = Dextrorse, 2 = Sinistrorse	2	2	0	2	0	0	0
T4: Stem cross‐section	1 = Diamond, 2 = Circular	2	2	0	2	0	0	0
T5: Stem color	1 = Green, 2 = Purple	2	1	1	1.64	0.48	29.37	0.66
T6: Stem tendril wings	1 = Empty, 9 = Exist	1	1	0	1	0	0	0
**T7: Stem anthocyanin color intensity**	1 = None or Extreme weak, 3 = Mild, 5 = Medium, 7 = Intense	7	1	6	4.43	2.02	45.5	1.33
T8: Stem thorn	1 = Empty, 9 = Exist	1	1	0	1	0	0	0
T9: Phyllotaxy	1 = Opposite, 2 = Alternate, 3 = Opposite and Alternate	3	3	0	3	0	0	0
**T10: Leaf shape**	1 = Heart‐shaped, 2 = Ovate, 3 = Lanceolate	3	2	1	2.04	0.21	10.06	0.18
T11: Leaf texture	1 = Leathery, 2 = Papery	1	1	0	1	0	0	0
T12: Number of leaf veins	Strip	7	5	2	6.91	0.41	5.95	0.18
T13: Leaf vein anthocyanin coloration	1 = Empty, 9 = Exist	1	1	0	1	0	0	0
**T14: Leaf color**	1 = Yellow‐green, 2 = Pale green, 3 = Medium green, 4 = Dark green	4	2	2	3.33	0.56	16.7	0.82
T15: Leaf apex	1 = Acuminate, 2 = Caudate	1	1	0	1	0	0	0
T16: Leaf base	1 = Lanceolate, 2 = Sagittate, 3 = Auriculate, 4 = Cordate	4	1	3	2.37	0.84	35.56	0.94
**T17: Leaf base color**	1 = green, 2 = purple, 3 = Purple red	3	1	2	1.7	0.51	30.21	0.75
T18: Petiole color	1 = green, 2 = pale green, 3 = purple, 4 = Purple‐green alternating	4	1	3	1.73	1.14	65.99	0.99
T19: Petiole anthocyanin coloration	1 = Empty, 9 = Exist	9	1	8	2.98	3.45	115.81	0.56
T20: Petiole tip color	1 = Purple, 2 = Green, 3 = Purple red	3	1	2	1.44	0.56	39.06	0.82
T21: Leaf incision	1 = Entire, 2 = Shallow, 3 = Medium, 4 = Deep	3	1	2	1.72	0.54	31.45	0.8
T22: Bulbil	0 = Empty, 1 = Exist	1	0	1	0.97	0.16	16.51	0.12
**T23: Number of bulbils**	1 = None or minimal, 3 = Small quantities, 5 = Medium quantity, 7 = Large quantity	7	1	6	6.04	1.53	25.33	0.92
T24: Degree of brown coloration of bulbil skin	1 = Empty, 3 = Superficial, 5 = Medium, 7 = Profound	7	1	6	4.22	1.58	37.52	1.1
T25: Flower	0 = Empty, 1 = Pistillate flowers, 2 = Male flowers	2	0	2	1.27	0.78	61.32	1.04
T26: Resistant to anthrax	1 = Nonresistant, 3 = Low‐resistant, 5 = Medium resistance, 7 = High resistance, 9 = Extreme‐resistant	7	1	6	3.58	1.61	44.96	1.19
T27: Resistant to brown spot	1 = Nonresistant, 3 = Low‐resistant, 5 = Medium resistance, 7 = High resistance, 9 = Extreme‐resistant	7	1	6	3.58	1.61	44.96	1.19
T28: Stem thickness	3 = Fine, 5 = Medium, 7 = Coarse	7	3	4	5	1.1	21.941	0.82
T29: Leaf base notch depth (cm)	3 = Shallow, 5 = Medium, 7 = Deep	7	3	4	4.95	0.68	13.67	0.43
T30: Leaf base notch width (cm)	1 = Narrow, 2 = Medium, 3 = Wide	3	1	2	1.97	0.51	26.32	0.76
T31: Leaf edge notch extent (cm)	1 = None or extreme‐weak, 3 = Mild, 5 = Medium, 7 = Intense	7	1	6	3.02	1.79	59.47	1.24
**T32: Leaf length (cm)**	3 = Short, 5 = Medium, 7 = Long	7	3	4	5	1.22	24.39	0.92
**T33: Leaf width (cm)**	3 = Narrow, 5 = Medium, 7 = Wide	7	3	4	4.96	1.25	25.13	0.94
T34: Leaf blade length‐to‐width ratio	1 = Elongated, 2 = Intermediate, 3 = Flat	3	1	2	2.03	0.43	21.23	0.61
T35: Petiole length (cm)	3 = Short, 5 = Medium, 7 = Long	7	3	4	5.04	0.7	13.96	0.46
**T36: Tuber cross‐section shape**	1 = Elliptical, 2 = Circular, 3 = Irregular	3	1	2	1.25	0.59	47.12	0.57
**T37: Tuber longitudinal‐section shape**	1 = Linear, 2 = Extreme‐narrow rectangle, 3 = Narrow rectangle, 4 = Extreme‐elliptical, 5 = Circular, 6 = Inverted triangle, 7 = Wide inverted triangle, 8 = Palm‐shaped, 9 = Other	9	1	8	3.57	2.65	74.41	1.12
T38: Tuber surface color	1 = Red, 2 = Yellow‐brown, 3 = Light brown, 4 = Medium brown, 5 = Dark brown, 6 = Purple, 7 = Black	3	2	1	2.04	0.21	10.06	0.09
T39: Tuber bud eye color	1 = Brown, 2 = Purple	1	1	0	1	0	0	0
T40: Tuber flesh color	1 = White, 2 = Cream, 3 = Orange, 4 = Magenta, 5 = Light purple, 6 = Medium purple, 7 = Dark purple	1	1	0	1	0	0	0
T41: Tuber length/cm	3 = Short, 5 = Medium, 7 = Long	7	3	4	5.12	1.28	25.06	0.96
**T42: Tuber width/cm**	3 = Narrow, 5 = Medium, 7 = Wide	7	3	4	4.84	1.25	25.86	0.94
T43: Tuber neck length/cm	3 = Short, 5 = Medium, 7 = Long	7	3	4	5.03	0.75	14.93	0.51
**T44: Tuber weight per plant/kg**	3 = Light, 5 = Medium, 7 = Heavy	7	3	4	5.04	0.92	18.29	0.66
T45: Bulbil weight/g	1 = Empty, 3 = Light, 5 = Medium, 7 = Heavy	7	1	6	3.97	1.16	29.28	0.88
**T46: Bulbil length/mm**	1 = Empty, 3 = Short, 5 = Medium, 7 = Long	7	1	6	5.85	1.33	22.65	0.87
**T47: Bulbil width/mm**	1 = Empty, 3 = Narrow, 5 = Medium, 7 = Wide	7	1	6	5.8	1.32	22.74	0.88
T48: Bulbil shape	1 = Elliptical, 2 = Spherical, 3 = Elongated, 4 = Irregular, 5 = Empty	5	1	4	1.5	0.8	53.46	0.85
T49: Tuber flesh viscosity	1 = Low, 2 = Medium, 3 = High	3	1	2	1.91	0.49	25.63	0.72
T50: Tuber flesh hardness	1 = Weak, 2 = Medium, 3 = Strong	3	1	2	2.3	0.53	23.04	0.78

*Note:* Core traits were indicated in bold.

### 2.2 DNA Extraction and SSR Fingerprinting

Previously, we used a yam transcriptome dataset to construct and annotate 181,047 unigenes (Li et al. 2020). Specific SSR primers were designed based on these unigene sequences using Primer3 (Li et al. 2022). Genomic DNA was extracted from young leaves in the vegetative growth stage of six selected individuals for each accession using a modified CTAB method (Allen et al. 2006). Targeted DNA fragments were amplified by 50 markers out of 100 SSR primer pairs, and five of those pairings exhibited clear polymorphisms. Besides, 14 SSR markers used by Yang et al. (2022) and Li et al. (2025) were selected for genotyping these yam accessions. Each SSR forward primer was fluorescently labeled with one of three dyes: HEX, 6‐FAM, and TAMRA. All primers were synthesized by Sangon Biotech (Shanghai, China).

PCR and thermal cycling parameters have been described in a previous study (Cao et al. 2021). The PCR amplification products were detected by 1.5% agarose gel electrophoresis. Subsequently, 10 μL of the liz500 mixture (liz500:HiDi = 1:99) was added to each PCR product, which was then incubated at 98°C for 5 min and immediately cooled on ice. Fluorescence detection was performed using an ABI 3730xl genetic analyzer (Applied Biosystems) by Sangon Biotech Co.

### 2.3 Data Analysis

The phenotypic data of 35 qualitative traits were categorized and assigned different values in accordance with Table 1. The frequency distribution of each class was determined, and the Shannon–Weaver diversity index (H′) was computed from the scores using the formula $H^{\prime }=-\sum_{i=1}^{n}\text{PilnPi}$, where Pi represents the relative frequency of accessions with the specific trait score (Kouam et al. 2018). Descriptive statistics, including the maximum, minimum, average, standard deviation (SD), and coefficient of variation (CV), were computed for 15 quantitative traits using SPSS 26.0 software. Principal component analysis (PCA) and the graphical representation of both DUS and SSR fingerprints were performed using R (v4.3.0). SSR marker genotypes were determined by using ABI GeneMapper software (v5.0). The raw data were processed by comparing the position of each peak with the Rox‐500 molecular weight internal standard within the corresponding lane to determine the fragment size of SSR markers. If only a single peak was detected at a locus, the genotype was recorded as homozygous. Alleles were General statistical parameters reflecting the genetic diversity of the 113 yam accessions, as summarized by Pagnotta (2018), were calculated using Popgene software (v1.32). These parameters included the number of alleles (*Na*), effective alleles number (*Ne*), observed heterozygosity (*Ho*), expected heterozygosity (*He*). Polymorphism information content (PIC) was determined using PIC‐CALC. All polymorphisms were assessed for the Hardy–Weinberg equilibrium to evaluate genetic variation and distribution. The UPGMA algorithm, as implemented within MEGA software (v4.1), was employed to construct a phylogenetic tree using genetic distances. The analysis of molecular variance (AMOVA) was conducted using GenAlEx (v6.51b2). Genetic structure analysis was conducted via Bayesian model‐based clustering implemented in STRUCTURE software v2.3.4 (Pritchard et al. 2000). For genotype clustering, the optimal number of clusters (*K*) was methodically tested from 1 to 10, with 10 independent iterations. Parameters for the burn‐in period and replication were configured at 100,000 and 200,000, respectively. The optimal *K* value was determined through Δ*K* analysis (Evanno et al. 2005) on STRUCTURE HARVESTER v0.6.94 (Earl and VonHoldt 2012). Following *K*‐value optimization, the CLUMPP_Windows 1.1.2 software (Jakobsson and Rosenberg 2007) was used to estimate the best membership coefficients for the accessions and DISTRUCT 1.1 (Rosenberg 2004) for the graphical representation.

## 3 Results

### 3.1 Phenotypic Diversity Analysis Based on DUS Testing

**FIGURE 2:**
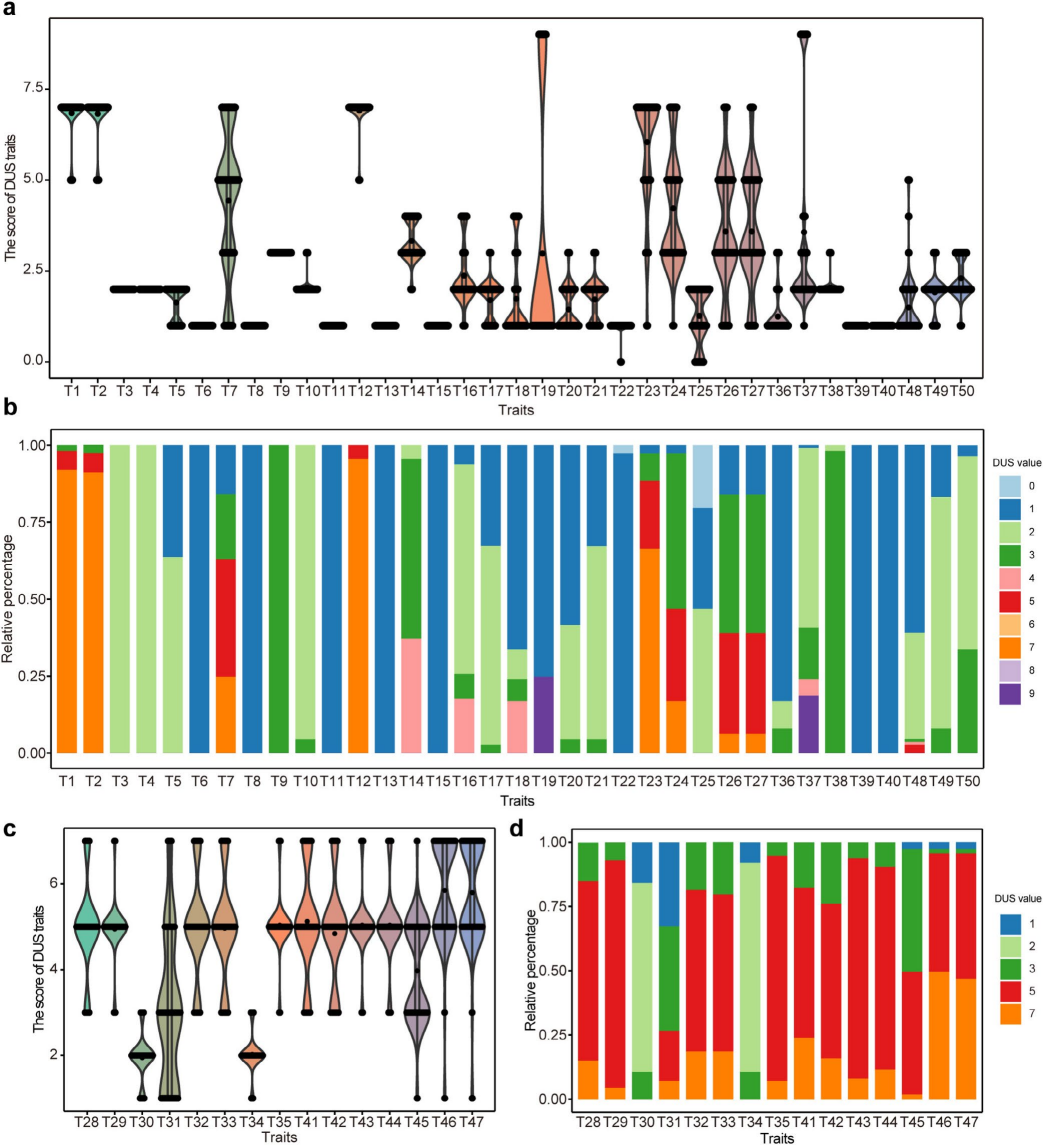
Variation in qualitative and quantitative traits among 113 yam accessions. (a) Box plots and (b) frequency distribution of 35 qualitative traits; (c) box plots and (d) frequency distribution of 15 quantitative traits. Trait codes T1 to T50 correspond to the 50 DUS traits evaluated, with full trait names provided in Table 1.

Fifty essential traits were selected from the Chinese yam DUS testing standard (NY/T 2495‐2013) based on growth and developmental stages, covering tissues including leaves, main plant stems, flowers, bulbils, and tubers. Among all samples, the average leaf shape varied from ovate‐orbicular to lanceolate (Figure 1b), bulbils showed continuous variation from spherical or ovoid to irregular (Figure 1c), while tubers demonstrated diversity in form, skin coloration, and texture (Figure 1d). Collectively, these morphological variations are critical for distinguishing different yam germplasm samples. Notable variability was observed across 104 landraces and 9 wild lines for 15 quantitative and 35 qualitative traits, based on the distribution of 50 DUS‐related traits (Figure 2). The majority of DUS qualitative traits exhibited substantial variation among the 113 yam cultivars. However, 10 traits showed no detectable phenotypic variation, including T3 (stem spirality), T4 (stem cross‐section), T6 (stem tendril wings), T8 (stem thorn), T9 (phyllotaxy), T11 (leaf texture), T13 (leaf vein anthocyanin coloration), T15 (leaf apex), T39 (color of the tuber bud eye), and T40 (color of the tuber flesh) (Figure 2a,b, Table S2). Parallel analysis of quantitative traits also indicated extensive genetic diversity, further supporting the presence of a rich genetic base within the yam germplasm resources (Figure 2c,d, Table S2). The Shannon–Weaver diversity index (H′) showed a range of 0–1.33, and the average was 0.61 (Table 1). The trait with the highest diversity was T7 (stem anthocyanin color intensity, H′ = 1.33), followed by T31 (leaf base notch extent, 1.24), T26 (resistant to anthrax, 1.19), and T29 (resistant to brown spot, 1.19). The 50 DUS traits demonstrated considerable differences in their coefficients of variation (CV%), with an average of 25.42% and a variation extent from 0% to 115.81% (Table 1). These patterns indicate substantial diversity in key traits among the tested materials, laying a groundwork for future variety identification.

**FIGURE 3:**
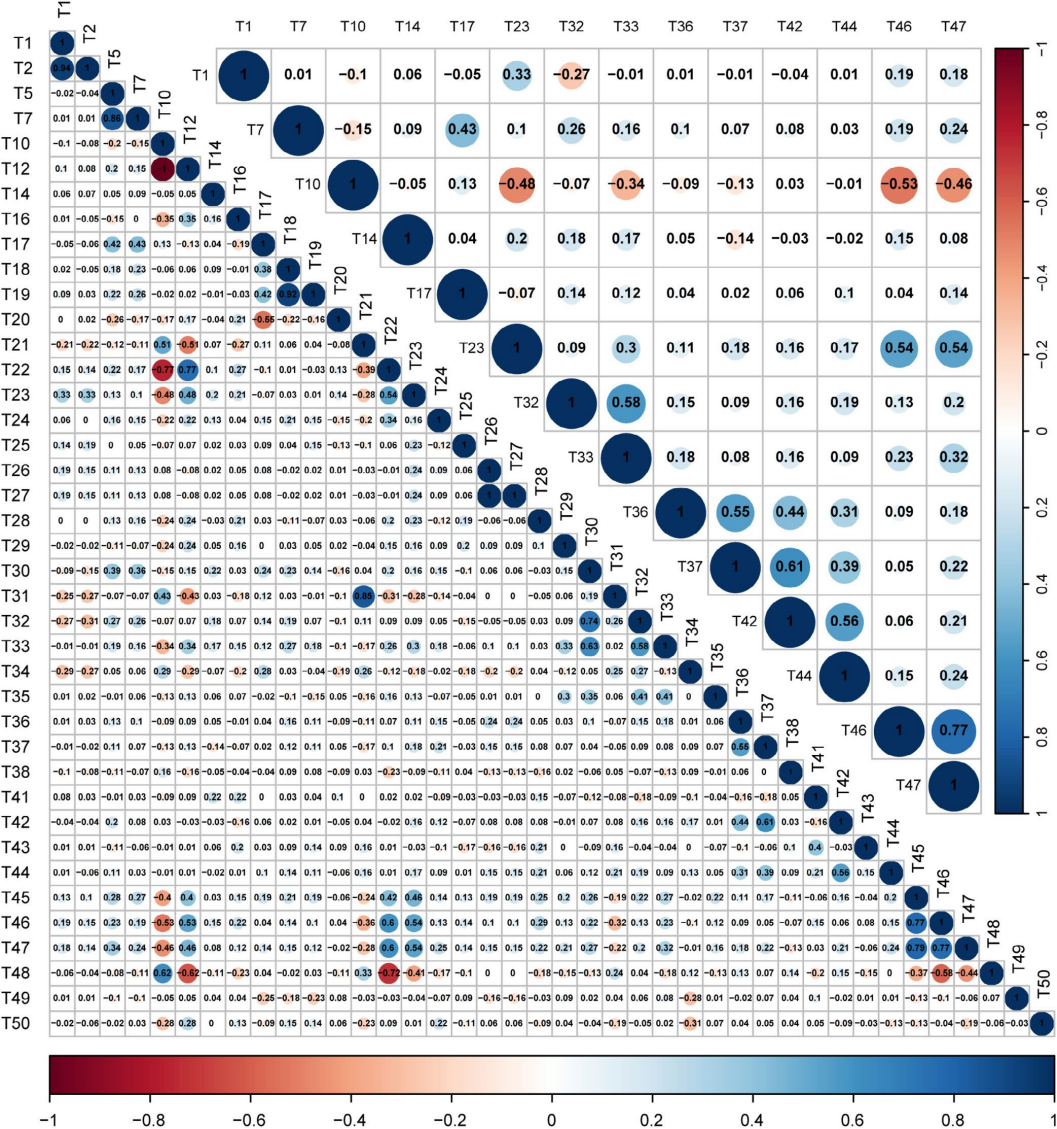
Correlation coefficients among 50 DUS testing traits (lower triangle) and 14 core traits (upper triangle) for accession identification in yam. The areas and colors of the circles show the absolute value of the corresponding correlation coefficients. Blue circles indicate positive correlations, and red circles indicate negative correlations. Values without background color indicate nonsignificant correlations (*p* > 0.01).

A pair‐wise correlation analysis of the remaining 40 assessed DUS traits revealed that each trait showed significant correlations with an average of three traits (Figure 3). T10 (leaf shape) and T12 (number of leaf veins) demonstrated significant correlations with 13 other DUS traits. Moreover, the traits T22 (bulbil), T23 (number of bulbils), T45 (bulbil quality), and T47 (bulbil width) exhibited significant correlations with 12 other traits. T33 (leaf width) was significantly correlated with 11 traits. T21 (leaf incision), T31 (leaf base notch extent), T34 (leaf blade length‐to‐width ratio), and T46 (bulbil length) were significantly correlated with 10 other traits. The correlation analysis revealed that leaf and bulbil traits are critical for the comprehensive DUS testing of yam varieties. By contrast, T14 (leaf color), T25 (flower), and T38 (tuber surface color) exhibited no significant correlation with other traits, implying these traits had minimal effects on the phenotypic expression of other traits.

### 3.2 Core Trait Selection and Performance

A core set of DUS traits was selected using PCA to optimize the DUS trait selection and assessment in the field. Twelve principal components with eigenvalues greater than 1 were extracted, and their cumulative contribution rate reached 76.31%, indicating that the first 12 principal components could represent the majority of the input information and serve as comprehensive indicators for evaluating yam phenotypic traits (Table S3). The first principal component (PC1) had the highest eigenvalue, 7.08, explaining 17.69% of the variation. As shown in Table S4, T47 (bulbil width), T12 (number of leaf veins), T22 (bulbil), and T46 (bulbil length) had high loading scores on PC1, suggesting that PC1 primarily represents the values of these indicators. PC2 exhibited an eigenvalue of 4.43 and explained 11.09% of the total variation in the germplasm collection. T30 (leaf base notch width) and T32 (leaf length) had high loading scores on PC2 (Table S4), indicating that PC2 primarily represented the values regarding leaf traits.

To calculate the principal component score, the weighting was based on the proportion of each principal component's eigenvalues to the total eigenvalues of the extracted principal components. The following comprehensive model was established: $\begin{array}{c} F=0.2318\times P1+0.1453\times P2+0.1047\times P3+0.0863\times P4+0.0764 \end{array}$ $\begin{array}{c} \times P5+0.0726\times P6+0.0626\times P7+0.0549\times P8+0.0477\times P9+0.0438 \end{array}$ $\begin{array}{c} \times P10+0.0384\times P11+0.0354\times P12 \end{array}$. Based on the PCA, correlation analysis between phenotypic traits and the *F* value was employed to refine the core traits (Table 2). The *F* value exhibited significant or highly significant positive correlations with 27 traits, and highly significant negative correlations with T10, T21, T31, and T48. Nine traits including T16, T20, T25, T34, T38, T41, T43, T49, and T50 showed no correlation with the *F* value. A stepwise regression equation was established based on the correlation analysis to screen the evaluation indicators. The obtained optimal model regression formula was as follows: $\begin{array}{c} Y=-6.056+0.177\times T47+0.238\times T44+0.149\times T33+0.159\times T7 \end{array}$ $\begin{array}{c} +0.137\times T23+0.11\times T36+0.27\times T46+0.155\times T1+0.159\times T32 \end{array}$ 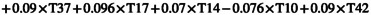. Regression analysis revealed a coefficient of determination (*R* ^2^) value of 0.984, indicating that merely 1.6% of the total variation was not explained by the model. Thus, 14 traits were identified as the core set for yam DUS testing, composed of six leaf traits, one stem trait, three bulbil traits, and four tuber traits (Table 1).

**TABLE 2:** Correlation coefficient between 40 phenotypic traits and the comprehensive value (*F*) in 113 yam germplasms.

Traits	Correlation coefficient	Traits	Correlation coefficient
T1	0.241*	T29	0.314**
T2	0.215*	T30	0.449**
T5	0.450**	T31	−0.197*
T7	0.426**	T32	0.450**
T10	−0.487**	T33	0.504**
T12	0.487**	T34	−0.098
T14	0.228*	T35	0.300**
T16	0.142	T36	0.458**
T17	0.276**	T37	0.416**
T18	0.336**	T38	−0.102
T19	0.288**	T41	0.047
T20	−0.144	T42	0.472**
T21	−0.289**	T43	−0.051
T22	0.533**	T44	0.465**
T23	0.619**	T45	0.689**
T24	0.271**	T46	0.706**
T25	0.141	T47	0.770**
T26	0.276**	T48	−0.381**
T27	0.276**	T49	−0.100
T28	0.245**	T50	−0.117

*Note:* *(*p* < 0.05) and **(*p* < 0.01) represent significant difference and extra significant difference, respectively.

The correlation coefficient matrix analysis of the 14 core traits revealed that each trait exhibited a significant correlation with two other DUS traits, comprising 41.9% of the total data points. In contrast, the corresponding value among the 40 investigated DUS traits was 29.1% (Figure 3). Particularly, T23 (number of bulbils), T46 (bulbil length), and T47 (bulbil width) demonstrated significant correlations with at least 10 core traits, all of which encompassed the bulbil (Figure 3). Therefore, these 14 traits can serve as effective screening indicators for yam germplasm evaluation, streamlining the morphological assessments, and improving breeding efficiency.

### 3.3 Cluster Analysis Based on Phenotypic Traits

**FIGURE 4:**
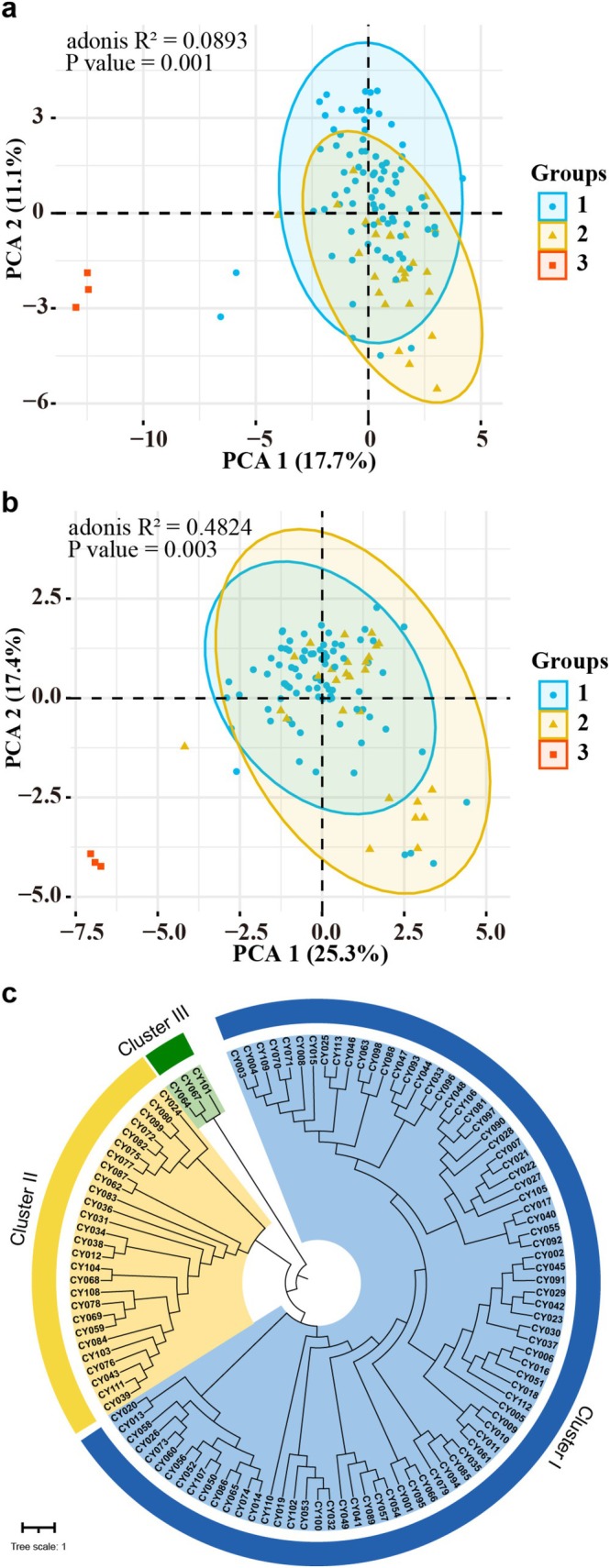
Principal component analysis (PCA) and cluster analysis of 113 yam accessions based on DUS traits. (a) PCA score plot derived from 40 DUS traits; (b) PCA score plot based on 14 core DUS traits; (c) cluster dendrogram constructed from phenotypic trait data.

Cluster analysis was executed for 113 yam assessed accessions based on the DUS traits investigated. PCA analysis conducted with the 14 core traits yielded similar results to the 40 DUS traits, which could be divided into three categories (Figure 4a,b). The dendrogram constructed from phenotypic traits revealed three major groups: Clusters I, II, and III (Figure 4c). Overall, 83 varieties were grouped in Cluster I, 27 in Cluster II, and 3 in Cluster III. These three clusters exhibited notable differences regarding the 14 core traits (Figure S1). Yam accessions in Cluster I exhibited significant differences from those in other clusters regarding T17 (leaf base color), exhibiting the thickest stems and the deepest leaf blade indentation. Cluster II accessions significantly differed from those in both Clusters I and III in T7 (stem anthocyanin color intensity) and T37 (tuber longitudinal‐section shape), with distinctive features including long and broad leaves, long petioles, wide leaf blade base indentation, heavy and large bulbils, as well as long and wide tubers with elongated tuber necks. Cluster III accessions exhibited significant differences from the other two clusters in traits T1, T7, T10, T14, and T23, exhibiting lower leaf density, weaker stem anthocyanin pigmentation, lanceolate leaves with five veins per leaf, pronounced leaf margin indentations, short petioles, slender stems, pale green leaf color, and absence of bulbils. It is noteworthy that among the three yam resources in Cluster III, none produced bulbils, whereas all 110 remaining germplasm resources contained bulbils (Table S2). Therefore, the presence or absence of bulbils may serve as a potential diagnostic criterion for identifying Cluster III.

### 3.4 SSR Marker Polymorphism Analysis

To evaluate genetic diversity, 19 SSR markers were assessed for 113 yam germplasm samples (Table S5). All markers generated distinct and stable bands and could be effectively used to evaluate the genetic variation of yam accessions (Figure S2). Partial amplification results were shown in Figure S3. The SSR marker alleles were arranged in alphabetical order according to amplified fragment size (Table S6). The polymorphic primer pairs resulted in 118 polymorphic alleles, averaging 6.21 alleles per SSR locus (Table 3). At each locus, the number of alleles (*Na*) ranged from 4 (Do2, SSR183, SSR214, SSR226, SSR362, and SSR377) to 15 (SSR275). The effective number of alleles (*Ne*) ranged from 2.0068 (SSR377) to 4.7293 (SSR275), with an average of 2.8106. The observed heterozygosity (*Ho*) ranged from 0.2054 (Do2) to 1 (SSR277), averaging 0.8611. The expected heterozygosity (*He*) ranged from 0.5039 (SSR377) to 0.7921 (SSR275), with an average of 0.6302. Nei's (1978) gene diversity ranged from 0.5017 (SSR377) to 0.7886 (SSR275), averaging 0.6274. Shannon's information index (*I*) spanned from 0.7637 (SSR377) to 1.9531 (SSR275), with an average of 1.1760. The polymorphism information content (PIC) value of SSRs ranged from 0.3925 (SSR377) to 0.7696 (SSR275), with an average of 0.5599. Except for Do2, SSR226, SSR368, and SSR377, the other 15 SSR primer pairs had PIC values greater than 0.5, indicating high levels of polymorphism among the assessed SSR markers. The results of the Hardy–Weinberg equilibrium (HWE) test indicated that most germplasm groups showed significant deviation from HWE at the majority of loci (*p* < 0.05). Especially, all loci in Cluster I exhibited highly significant deviation from HWE (*p* < 0.01) (Table S7).

**TABLE 3:** Details of polymorphic markers along with their allelic diversity and PIC.

Marker	*Na*	*Ne*	*Ho*	*He*	Nei	*I*	PIC
Do2	4	2.0954	0.2054	0.5251	0.5228	0.9131	0.4608
Do22	6	2.6809	0.9115	0.6298	0.6270	1.1951	0.5710
Do7	8	2.5391	0.8761	0.6088	0.6062	1.1067	0.5268
SSR1	8	3.5314	0.9459	0.7201	0.7168	1.5555	0.6850
SSR13	6	4.1527	0.9820	0.7626	0.7592	1.5725	0.7224
SSR17	6	2.4968	0.9196	0.6022	0.5995	1.0654	0.5201
SSR183	4	2.6411	0.9273	0.6242	0.6214	1.0771	0.5457
SSR214	4	2.4354	0.9735	0.5920	0.5894	0.9928	0.5066
SSR226	4	2.3483	0.8053	0.5767	0.5742	0.9408	0.4808
SSR232	6	2.4322	0.9823	0.5915	0.5888	1.0245	0.5026
SSR275	15	4.7293	0.6460	0.7921	0.7886	1.9531	0.7696
SSR277	7	3.0859	1.0000	0.6790	0.6759	1.3582	0.6279
SSR362	4	2.7432	0.9640	0.6383	0.6355	1.0792	0.5609
SSR368	5	2.1659	0.9469	0.5407	0.5383	0.8751	0.4347
SSR373	9	3.1393	0.6822	0.6847	0.6815	1.3066	0.6214
SSR377	4	2.0068	0.6991	0.5039	0.5017	0.7637	0.3925
SSR41	5	2.5171	0.9643	0.6054	0.6027	1.0665	0.5303
SSR53	7	2.9799	0.9554	0.6674	0.6644	1.3283	0.6186
SSR92	6	2.6806	0.9732	0.6298	0.6270	1.1697	0.5608
Mean	6.2105	2.8106	0.8611	0.6302	0.6274	1.1760	0.5599
Total	118	53.4013	16.36	11.9743	11.9209	22.3439	10.6385

### 3.5 Genetic Diversity and Germplasm Structure of the 113 Yam Accessions

**FIGURE 5:**
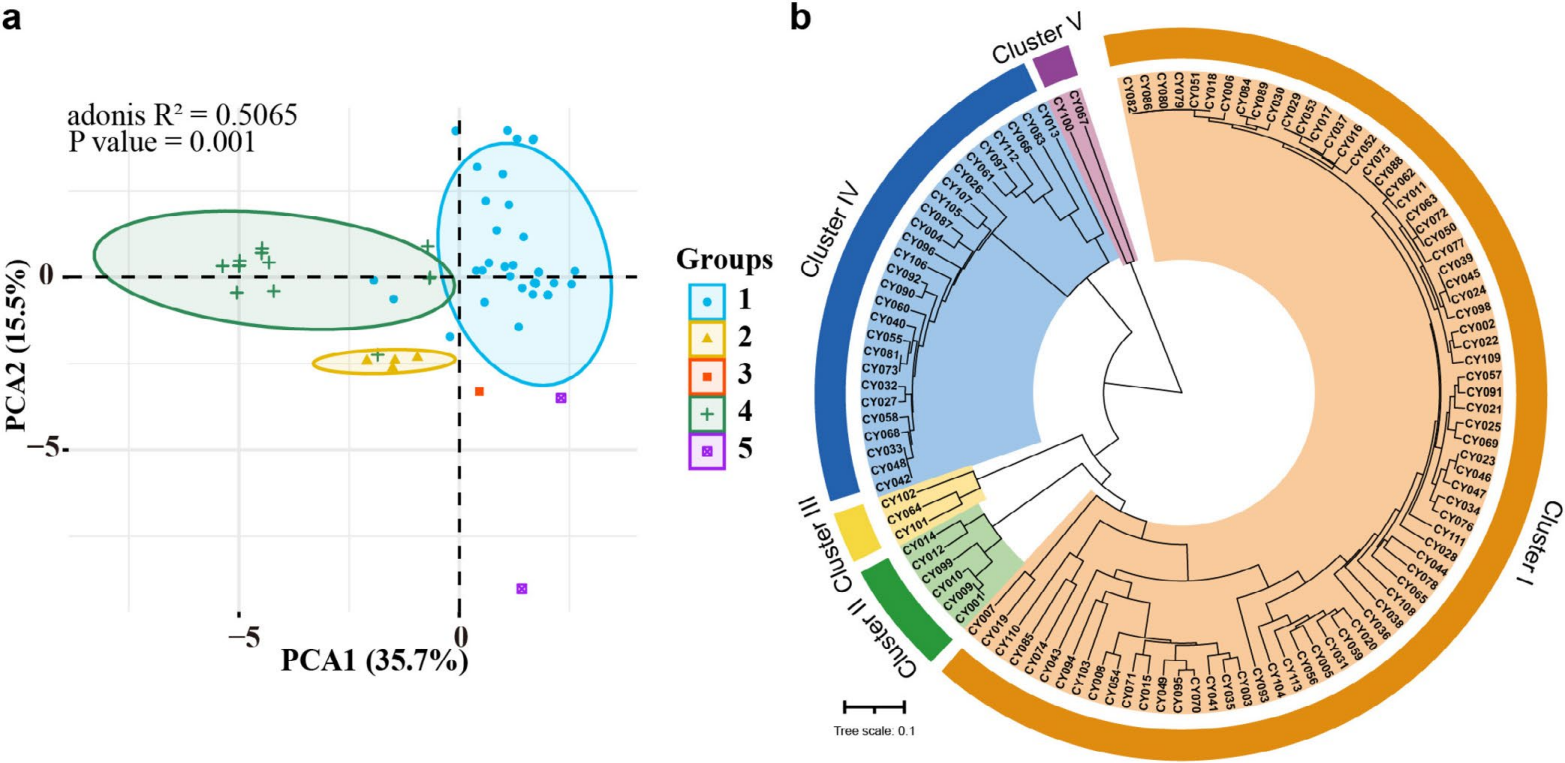
Genetic diversity analysis of 113 yam accessions using 19 SSR markers. (a) Principal component analysis (PCA) score plot; (b) UPGMA dendrogram illustrating genetic relationships based on Nei's genetic distance coefficient.

The 113 accessions were organized into five groups through the cluster analysis of SSR data (Figure 5, Figure S4). PCA using the pair‐wise genetic distance matrix supplemented the cluster analysis, grouping the samples into five clusters (Figure 5a). The first two PCA axes accounted for 35.7% and 15.5% of the overall variance, respectively. The genetic distance matrix of the 113 yam varieties was derived from the genotyping data of all 118 alleles. Cluster I consisted of 75 accessions, which represented 66.37% of the total germplasm. Cluster II contained 6 accessions (CY001, CY009, CY010, CY012, CY014, and CY099), accounting for 5.31% of the total germplasm samples evaluated. Cluster III comprised three accessions, namely CY064, CY101, and CY102, collectively representing 2.65% of the evaluated set. Cluster IV comprised 27 accessions, representing 23.89% of the total germplasm. Cluster V, comprising 2 wild accessions (CY067 and CY100), represented 1.77% of the investigated yam accessions. SSR fingerprinting revealed that cultivars with different names had identical genetic profiles, suggesting they were synonymous (Cluster I: CY082, CY086, CY080, CY079, CY051, and CY018; CY084 and CY089; CY029, CY053, and CY017; CY075, CY088, CY062, and CY011; CY063, CY072, and CY050; CY039, CY045, and CY024; CY023 and CY046. Cluster II: CY001 and CY009. Cluster IV: CY033, CY048, and CY042; CY073 and CY081) (Figure 5b). Nine groups of potentially redundant yam genotypes were identified among the 113 yam accessions using a genetic distance (GD) threshold of 0 and the SSR marker‐derived dendrogram. Finally, after eliminating the redundant genotypes (19 accessions), 94 distinct yam accessions were identified. On the contrary, certain germplasm samples with the same or similar name exhibited genetic dissimilarities, indicating they were homonymous (e.g., CY001, CY010, and CY070; CY002, CY005, and CY087; CY003 and CY004; CY078 and CY097; CY042 and CY111; CY072 and CY088; CY048, CY073, and CY106; CY028 and CY063; CY009 and CY012; CY013 and CY021; CY020 and CY067; CY062 and CY091; CY102 and CY104) (Figure 5b).

**FIGURE 6:**
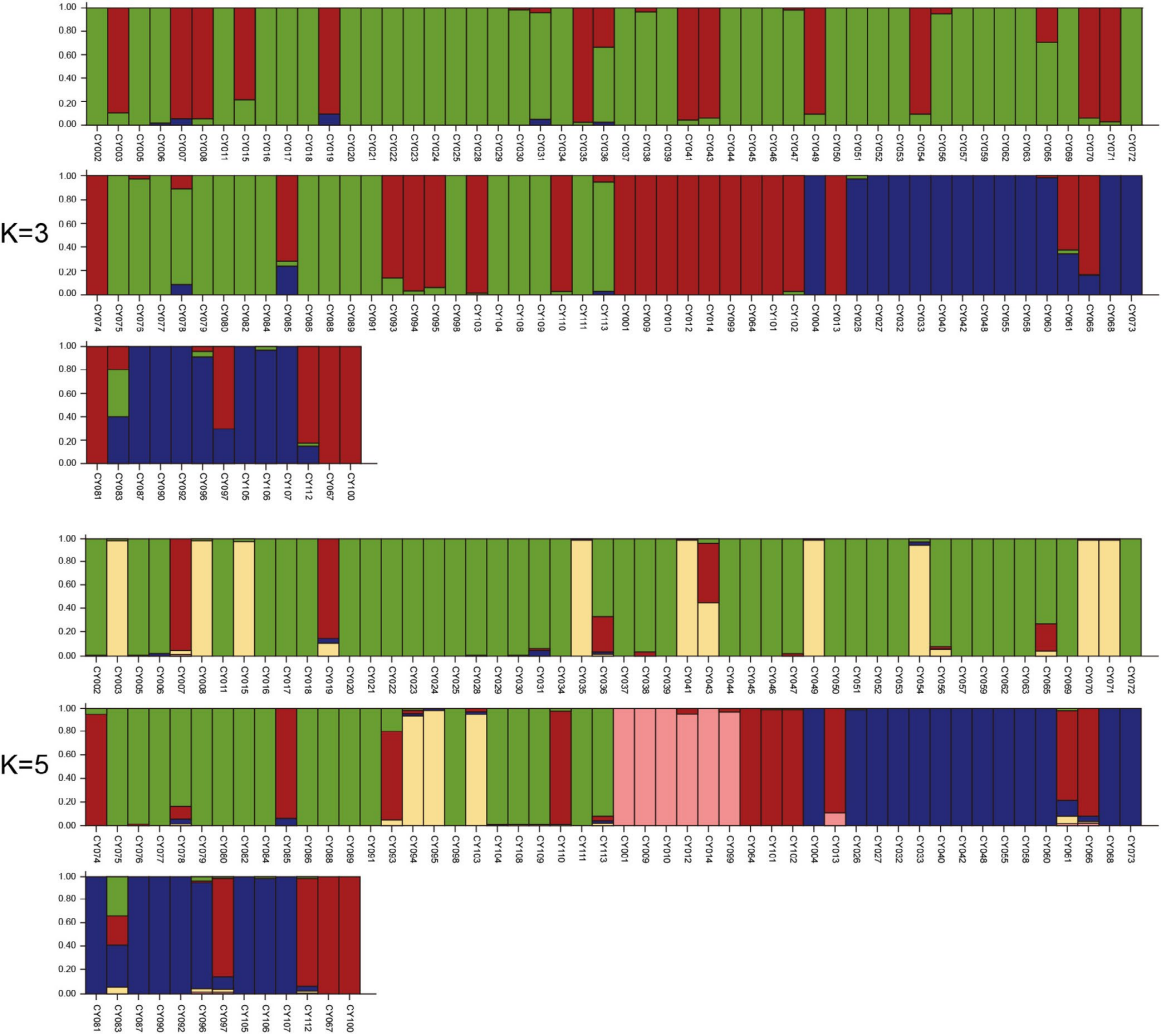
Genetic structure of 113 yam accessions at *K* = 3 and *K* = 5 based on 19 SSR markers. Each accession is represented by a vertical bar, with numbers corresponding to Table S1.

The genetic structure of 113 yam accessions was examined using STRUCTURE v2.3.4 to further support the results of phylogenetic and PCA analyses (Pritchard et al. 2000). According to STRUCTURE HARVESTER, the optimal number of genetic clusters was *K* = 3, which yielded the highest Δ*K*. Although slightly lower, the Δ*K* value at *K* = 5 was also notably high, indicating a well‐supported, finer genetic structure (Figure S5). At *K* = 3, the 113 accessions were classified into three groups: Group I (designated in green), Group II (red), and Group III (blue) (Figure 6). We subsequently compared the structure analysis results and phylogenetic tree clustering. Group I was the largest, comprising a collection of 56 accessions from Cluster I. Group II (35 accessions) contained 19 accessions from Cluster I and 5 accessions from Cluster IV, along with all accessions from Clusters II, III, and V. Group III comprised 22 accessions from Cluster IV. At *K* = 5, the genetic structure showed further refinement, revealing five distinct groups (Figure 6). Group I remained consistent with the 56 accessions from Cluster I. Group II was reduced to 16 accessions, including 6 from Cluster I, 5 from Cluster IV, and all members of Clusters III and V. Group III was formed solely by 13 accessions from Cluster I. Notably, Group IV distinctly separated all accessions of Cluster II, suggesting a unique genetic background for this group. Group V corresponded to the 22 accessions from Cluster IV. A certain degree of genetic exchange occurred between groups, aligning with the findings of the UPGMA‐based dendrogram and PCA analysis. Although the peak ΔK at *K* = 3 represents the most significant level of genetic division, the structural details observed at *K* = 5 remain biologically meaningful, indicating further genetic differentiation within the three major groups and demonstrating a hierarchical genetic architecture in the germplasm.

The patterns of variance within the germplasm collection were assessed using molecular variance (AMOVA) analysis based on data obtained from SSR markers. The majority of genetic variation (82.12%) occurred within genetic groups, with the rest (17.87%) being among them (Table 4). In addition, the overall *F*_ST_ value (0.32) demonstrated a very high level of genetic differentiation across the 113 yam accessions. The average gene flow (Nm) among the accessions was 0.52 (Table 4).

**TABLE 4:** Analysis of molecular variance (AMOVA) of 113 yam accessions.

		Degree of freedom (df)	Square sum (SS)	Mean square (MS)	Estimated variance (Est. Var.)	Percentage of total variation
Source	Among groups	4	207.4946	51.87364	1.763481	17.8757%
	Among individual	108	237.563	2.199657	0.000	0
	Within individual	113	915.500	8.10177	8.10177	82.1243%
	Total	225	1360.558		9.865251	100%
*F*‐statistics	*F*_ST_	0.3229				
	Nm	0.5241				

### 3.6 Comparison of DUS Testing and SSR Genotyping for *D. polystachya* Germplasm Identification

**FIGURE 7:**
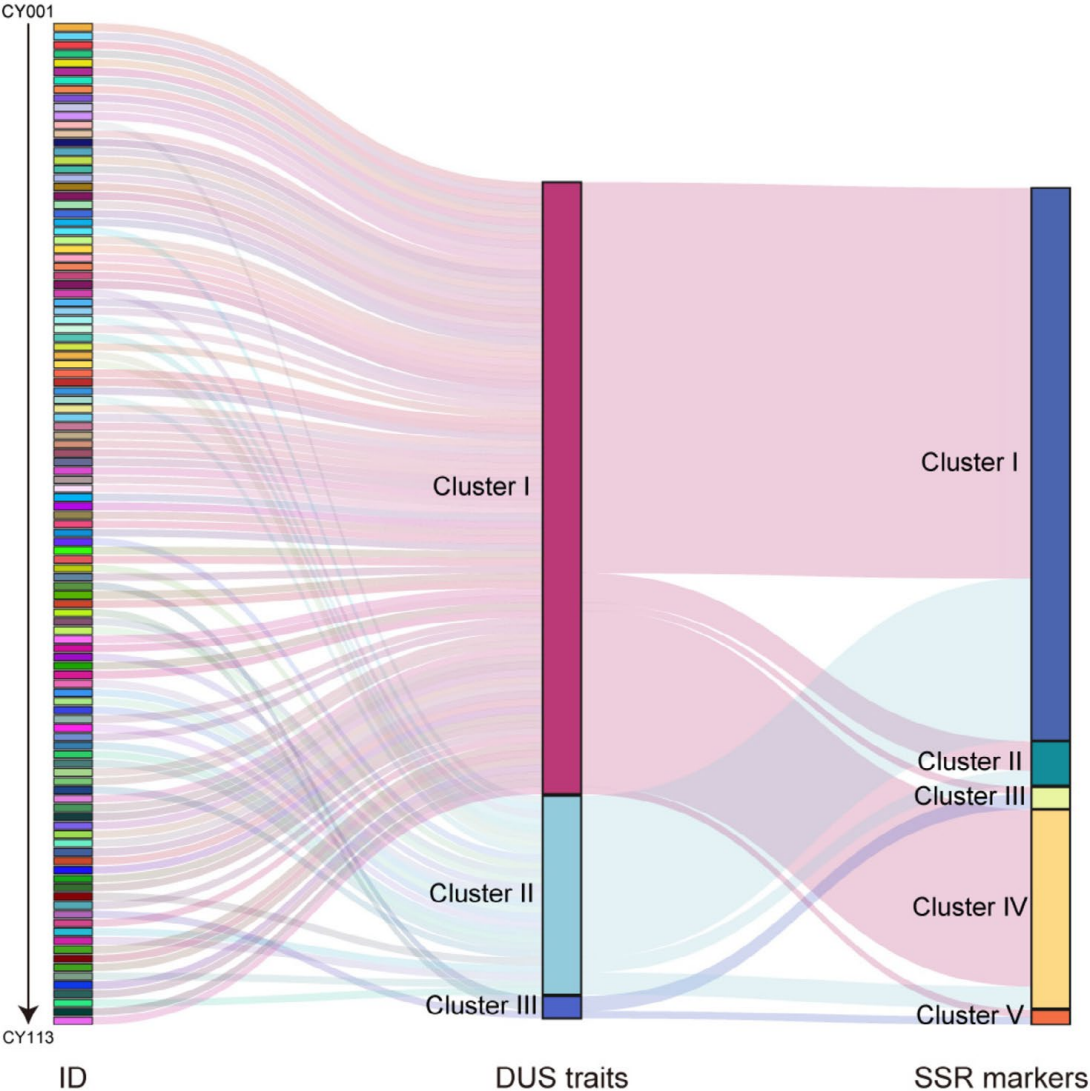
Correspondence of germplasm accessions identification between DUS traits and SSR genotyping. Three yam accessions.

Both morphological markers (based on DUS testing) and SSR molecular markers are effective in revealing the genetic diversity of yam germplasm resources and in distinguishing most of the tested accessions. Morphological clustering grouped the germplasms into three categories with distinct phenotypic characteristics, whereas SSR markers delineated five genetic groups. Since molecular markers reflect genomic‐level variation, some discordance between the two methods was observed. Specifically, SSR‐based Group I comprises 75 accessions, including 53 from morphological Class I and 22 from Class II. Group II consists of 6 accessions, with 4 originating from morphological Class I and 2 from Class II. Group III includes 3 accessions, among which 2 belong to morphological Class III and 1 to Class I. Group IV contains 27 accessions, with 24 from morphological Class I and 3 from Class II. Group V consists of two accessions: one from morphological Class I and one from Class III. Both are wild yam accessions that are morphologically distinct from other varieties, characterized by five‐veined, lanceolate, and deeply lobed leaves (Figure 7, Table S8).

## 4 Discussion

Most yam varieties are propagated asexually and exhibit variable ploidy levels and complex genetic backgrounds. They also exhibit considerable morphological intravarietal and intervarietal differences (Darkwa, Olasanmi, et al. 2020). Accurate identification of yam varieties is crucial for fostering breeding innovations, safeguarding the interests of growers, and ensuring food safety in agricultural production. Currently, phenotypic and genotypic assessments based on DUS testing standards and SSR markers are commonly used to study genetic diversity and identify individual genotypes. In this study, the authenticity of 113 *D. polystachya* varieties was verified using 50 DUS testing traits and 19 SSR markers.

### 4.1 Assessment of Genetic Diversity in *D. polystachya* Based on Phenotypic Traits

For 15 quantitative traits, the coefficients of variation ranged from 11.81% to 105.26%, with an average of 31.11%. The coefficient of variation was highest for leaf edge indentation and lowest for the leaf length‐to‐width relationship. This finding differs from the results of Cao et al., where among the six quantitative traits assessed in *D. opposita* , the tuber flesh weight displayed the highest CV (Cao et al. 2021). The discrepancy may be due to differences in the yam germplasm selection and the quantitative traits examined. As defined previously, a trait's variation is considered significant when the coefficient of variation surpasses 10% (Zhang et al. 2017). The CV% values for nine traits, including stem thickness, leaf base notch depth, leaf edge indentation degree, weight of bulbils, length of bulbils, width of bulbils, tuber width, tuber neck length, and tuber weight per plant, were greater than 20%, indicating a rich diversity within yam germplasm. The Shannon–Wiener index of genetic diversity spanned from 0.43 to 1.24 in the 113 yam accessions. Among the traits that were assessed, the leaf edge indentation exhibited the highest diversity index. Stem anthocyanin color intensity had the largest diversity index among 35 quality traits. Overall, quantitative traits exhibited a higher average diversity index compared to qualitative traits, indicating a richer genetic diversity in quantitative traits. This was consistent with the results of a morphological diversity analysis in lentil germplasm resources (Tripathi et al. 2022).

DUS testing is time‐consuming, labor‐intensive, costly, and susceptible to environmental influences (Jamali et al. 2019). To streamline this process, a core set of traits was selected via PCA based on initial eigenvalues > 1 and cumulative contribution rate (Zhang, Cao, et al. 2022). Although the first 12 principal components had eigenvalues > 1, their cumulative contribution reached only 76.31%, indicating multidirectional trait variation. Previous studies have also demonstrated substantial phenotypic diversity in yam using similar approaches (Adjei et al. 2022; Zhang et al. 2025). In this study, 50 phenotypic traits were reduced to a core set of 14 traits through PCA and stepwise regression. Statistical analysis confirmed that these 14 traits effectively represented the diversity captured by the original 50 traits, supporting their use for efficient germplasm identification. Furthermore, morphological clusters showed breeding potential: Cluster I for thicker stems, Cluster II for heavier bulbils and longer/wider tubers with extended necks, and Cluster III for higher tuber weight per plant.

In previous studies, three traits (stem thorn, stem wing, and stem spirality) showed no variation in *D. polystachya* (Cao et al. 2021). However, unfortunately, these three traits are not unique and thus cannot serve as diagnostic features for distinguishing *D. polystachya* from *D. alata* , *D. persimilis* , *D. fordii* , and *D. esculenta* . In this study, we observed a lack of variation in a total of 10 traits, including these three previously reported monomorphic traits (Figure 2, Table S2). Further research is needed to investigate the performance of these traits in other similar species to identify potential unique traits specific to other *Dioscorea* species. Moreover, we found that certain potentially informative traits, such as those associated with seeds, are currently not included in the standard DUS test protocol. Among the three germplasm groups classified based on DUS traits, all accessions in the third group produced abundant seeds, while the other two groups produced few or none (data not shown). Therefore, refining phenotypic test traits of *D. polystachya* is critical and necessary for enhancing the analysis of genetic diversity rooted in phenotypic characteristics.

### 4.2 Assessment of Genetic Diversity in *D. polystachya* Through SSR Maker Analyses

SSR markers have been widely employed to estimate genetic diversity across different species (Cao et al. 2021; Hu et al. 2022; Li et al. 2022). The 19 SSR markers, which exhibited high polymorphism and effectively captured the genetic variation in *D. polystachya* , amplified a total of 118 alleles with an average of 6 alleles per locus (*Na*). The mean effective allele count was lower at 2.8 (Table 3), suggesting uneven allele frequencies at each locus. Cao et al. (2021) reported a slightly lower number of alleles in 112 yam accessions (*Na* = 4, *Ne* = 2.5). Conversely, a higher number of alleles was reported in a previous study in Guinea yam (*Na* = 8.69) (Loko et al. 2017). The main reason for this discrepancy may be due to the differences in SSR primers employed and the yam germplasm resources examined. We utilized SSR markers specific to *D. polystachya* , which could more effectively elucidate the genetic diversity and phylogenetic relationships within *D. polystachya* germplasm resources.

The genetic diversity evaluation of these varieties revealed PIC and *I* values of 0.5599 and 1.176, which were higher than earlier reports (Cao et al. 2021; Wu et al. 2019). Shannon's indices reflect genetic diversification, wherein the larger the value, the higher the dispersion degree within the group, and the richer the diversity (Siqueira et al. 2014). The PIC value, which reflects the discriminating ability of a marker and is determined by the number of known alleles and their frequency distribution, is not affected by factors such as geographical origin, the quantity of germplasm resources, or the gene loci investigated (Nachimuthu et al. 2015). The Hardy–Weinberg equilibrium (HWE) test is an effective method for studying genetic variation in a natural population, which predicts that gene frequencies and genotype frequencies in diploid populations vary (Stern 1943; Wang and Shete 2012). In this study, the HWE test results showed significant deviation from expectations (Table S7), which may be attributed to the polyploid nature of *D. polystachya* . Although the ploidy level of *D. polystachya* has not been explicitly documented, it is hypothesized that members of the *Dioscorea* genus underwent an ancient paleo‐tetraploidization event during their evolutionary history (Bredeson et al. 2022).

During the long‐term domestication and cultivation of yam, a series of local varieties has been developed. However, few have been officially registered, and the lack of a unified naming standard for introduction and promotion across regions has resulted in nomenclatural confusion among varieties. Through SSR marker‐based genetic diversity analysis of yam, we identified potential cases of synonymy and homonymy, laying a foundation for the effective development and utilization of yam germplasm resources.

### 4.3 Comparison of Phenotypic Traits and SSR Markers in Genetic Diversity Analysis

Based on DUS testing, the grouping pattern revealed that the 113 yam accessions could be categorized into three distinct groups. However, this finding did not align with the results obtained from fingerprinting the 19 SSR markers in the same set of accessions. Despite the distinct differences in the clustering patterns generated by the two methods, notable similarities are also apparent. The disparities observed may be due to the different methods of clustering. Clustering in DUS trait testing is predicated on morphological traits, which are significantly influenced by genetics and environmental factors such as cultivation practices, soil attributes, and nutrient management, while SSR marker‐based clustering relies on individual marker loci (Cao et al. 2021; Darkwa, Agre, et al. 2020). Unlike morphological markers, molecular markers are stable across environments (Premjet et al. 2022). SSR molecular markers are considered to be one of the most effective molecular fingerprinting methods, providing efficiency and accuracy in yam variety identification (Chen et al. 2022; Diouf et al. 2024; Wang, Wang, et al. 2022). It is also possible that the primers utilized in this study do not comprehensively encompass all loci influencing morphological traits, and these marker loci may not completely correlate with these traits. *F*_ST_ values indicate little (< 0.05), moderate (0.05–0.15), strong (0.15–0.25), or very strong (> 0.25) genetic differentiation (Cho et al. 2011). In our study, the *F*_ST_ value across the entire set of accessions was high, indicating significant genotype variability (*F*_ST_ = 0.3229, Table 4), suggesting that these accessions could be valuable for yam cultivar breeding programs. Genetic structure and PCA analysis categorized the yam accessions into three and five groups, respectively. The discrepancies between genetic structure assessment and phylogenetic tree clustering might be due to variations in the unique algorithms. These findings differ from those of a prior study based on SSR‐SRAP data, which categorized 112 *Dioscorea* spp. accessions into six groups (Cao et al. 2021). Such disparities might be attributed to the distinct yam accessions and primers utilized in each of the respective studies. Our study incorporated a larger sample of 113 *D. polystachya* accessions, which more comprehensively represented the genetic diversity of *D. polystachya* across various regions and ecological environments. Meanwhile, we used species‐specific primers for *D. polystachya* to enhance the accuracy and reliability of the molecular fingerprinting of the germplasm. Future studies could further enhance the precision of molecular markers and the information obtained by increasing the number of primers utilized. Notably, morphological clustering grouped Taihangshan wild yam (CY067) with Dengxianfeng yam (CY064) and Jiujinhuang yam (CY101) into one group, and Xiuwu qinglongxia wild yam (CY100) with Nanjing cai yam (CY102) into another group (Figures 4 and 5), failing to distinguish wild accessions from cultivated accessions, indicating that SSR molecular markers are more suitable for distinguishing wild yam resources from cultivated yam varieties.

Germplasm resources underpin crop breeding, and exploiting superior traits drives breeding progress. This study analyzed 113 yam accessions using phenotypic traits and SSR markers, revealing low direct correlation or consistency between methods. Although traits are genetically controlled, phenotypic expression is also influenced by environmental conditions. Furthermore, the selected molecular marker loci may not align with genomic regions responsible for trait expression, contributing to the observed discrepancies between phenotypic and molecular assessments. Agronomic traits serve as the foundation for classification and diversity analysis, playing an indispensable role in field‐based screening, variety registration, and on‐farm identification. In contrast, molecular markers provide critical support for genetic identity verification, pedigree analysis, and elucidating population structure. To improve the accuracy of yam evaluation and parental selection, phenotypic and molecular methods should be integrated through broadening phenotypic trait sets and developing new primers, which will enhance germplasm characterization and diversity assessment.

### 4.4 Future Prospects for Variety Identification in Plant Breeding

*Dioscorea polystachya* are generally propagated vegetatively through their tubers due to the absence of fertile and seed‐setting genotypes. As a result, germplasm introduction is the only reliable method for acquiring better yam varieties (Darkwa, Olasanmi, et al. 2020; Mondo et al. 2022). However, the frequent and extensive introduction of *D. polystachya* cultivars from diverse regions leads to major issues with homonymous and synonymous yam varieties. Using SSRs or other molecular markers to analyze genetic variation and relatedness among diverse yam accessions helps identify phenotypes, detect redundant accessions, and build core germplasm panels, and this approach may reduce maintenance costs and support the selection and breeding of high‐quality yam cultivars (Adjei et al. 2023; Cao et al. 2021; Diouf et al. 2024). In‐depth analyses of the phenotypic data will be critically important to applications in plant breeding. Therefore, fully excavating the phenotypic trait information combined with molecular marker data will greatly facilitate the establishment of a core germplasm repository and the collection of germplasm resources. Moreover, as a traditional Chinese medicinal herb with both edible and medicinal values, quality characteristics are important indicators for evaluating the superiority of yam varieties and screening high‐quality healthcare varieties. Establishing genetic diversity analysis based on quality indicators can also provide references for screening excellent yam germplasm resources with high yield and good quality.

Distinctly from other studies, our research aims to elucidate the variations in genetic diversity among different *D. polystachya* varieties while systematically resolving the ambiguities regarding these varieties used in agricultural production. Notably, this study successfully identified 10 groups of potential heterotypic synonyms and 13 groups of potential homonyms among 113 accessions of *D. polystachya* using a genotyping matrix based on 19 SSR markers. This demonstrates the effectiveness and benefits of these specific SSR markers in identifying and authenticating germplasm resources among different varieties within *D. polystachya* . Moreover, this study offers valuable phenotypic data for locating the genes responsible for key agricultural traits in yam.

## 5 Conclusion

Overall, the assessed *D. polystachya* accessions exhibited substantial genetic diversity, with significant genomic variation among these genotypes. The 113 *D. polystachya* varieties collected from 17 provinces in China were clearly distinguished both by 19 informative SSR markers and 50 DUS traits. Additionally, a new set of 14 core DUS traits was selected, improving variety identification efficiency through simpler processing. Taken together, the newly selected SSR markers and core traits are highly valuable for yam genetic research. The assessment of genetic diversity among yam varieties will also furnish valuable information for the subsequent utilization of these yam germplasm resources in breeding initiatives.

## Author Contributions

**Jin Gao:** formal analysis (equal), investigation (equal), software (equal), writing – original draft (equal), writing – review and editing (equal). **Jiangli Zhang:** conceptualization (equal), project administration (equal), supervision (equal). **Jiage Wang:** data curation (equal), investigation (equal). **Yingying Chang:** methodology (equal), visualization (equal). **Zhao Qin:** formal analysis (equal), methodology (equal). **Lintao Sun:** data curation (equal), visualization (equal). **Mingjun Li:** project administration (equal), supervision (equal). **Qingxiang Yang:** supervision (equal), validation (equal), writing – review and editing (equal).

## Conflicts of Interest

The authors declare no conflicts of interest.

## Supporting information


**Figure S1:** Box plot analysis showing variation in the 14 core DUS traits across three clusters. Clusters are color‐coded as follows: I (blue), II (yellow), and III (green). Full names of the traits are provided in Table 1.
**Figure S2:** Gel electrophoresis images of amplified alleles from SSR‐based molecular markers in random yam samples.
**Figure S3:** Electropherogram detected by Do7 for 2 yam accessions.
**Figure S4:** UPGMA phylogenetic tree illustrating genetic relationships among five groups, based on genetic distances derived from 19 SSR markers.
**Figure S5:** Genetic structure analysis in 113 yam accessions.

**Table S1:** Name of 113 yam accessions.
**Table S2:** Phenotypic data of 113 yam germplasm resources.
**Table S3:** Principal component analysis of 40 traits in 113 yam germplasms.
**Table S4:** Loading of factors in principal components.
**Table S5:** List of the 19 SSR markers selected.
**Table S6:** Genotyping matrix for 19 SSR markers.
**Table S7:** Hardy–Weinberg equilibrium at 19 microsatellite loci among 5 groups of *Dioscorea polystachya* .
**Table S8:** Germplasm classification results by DUS testing and SSR in 113 yam accessions.

## Data Availability

The data that supports the findings of this study is available in the Supporting Information of this article.
