# Supplementary material for: Intraspecific Genetic Diversity Analyses of Yam (Dioscorea polystachya Turcz.) Based on DUS Traits and SSR Molecular Markers
Source: Ecol Evol. 2025 Oct 16;15(10):e72295. doi: 10.1002/ece3.72295 (PMC12530841; doi:10.1002/ece3.72295)
Supplement: Supplementary file 1 — Figure S1: Box plot analysis showing variation in the 14 core DUS traits across three clusters. Clusters are color‐coded as follows: I (blue), II (yellow), and III (green). Full names of the traits are provided in Table 1. Figure S2: Gel electrophoresis images of amplified alleles from SSR‐based molecular markers in random yam samples. Figure S3: Electropherogram detected by Do7 for 2 yam accessions. Figure S4: UPGMA phylogenetic tree illustrating genetic relationships among five groups, based on genetic distances derived from 19 SSR markers. Figure S5: Genetic structure analysis in 113 yam accessions. [file ECE3-15-e72295-s002.docx]

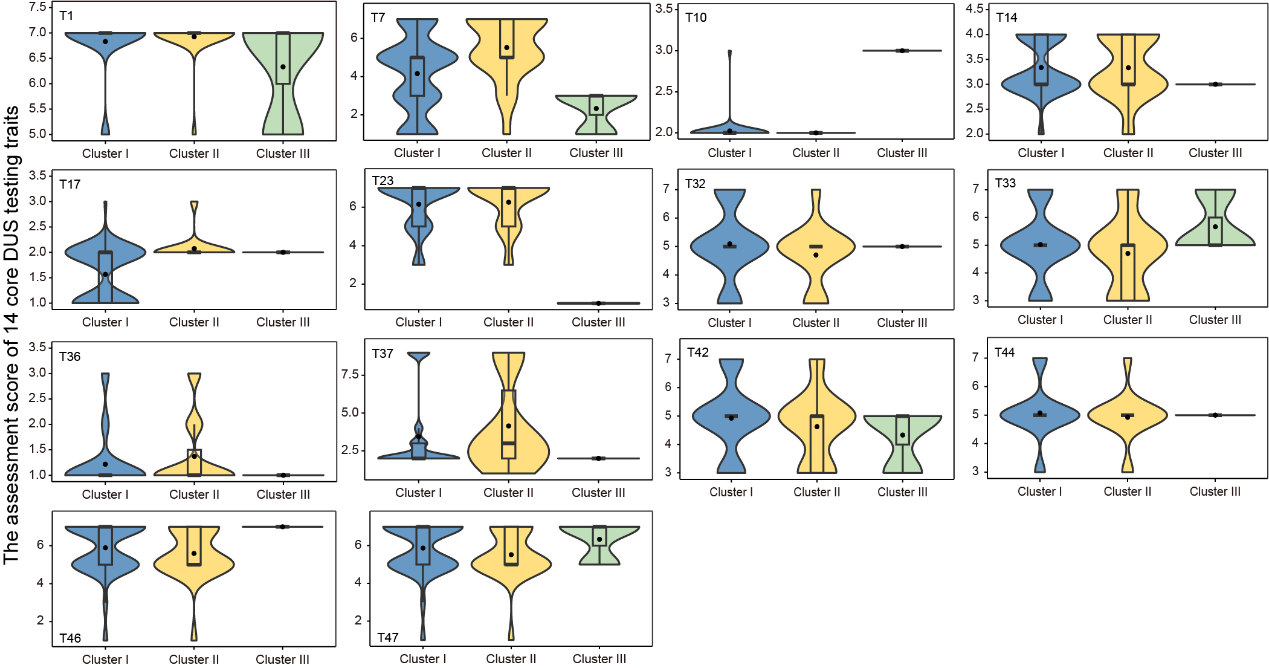


Figure S1. Box plot analysis showing variation in the 14 core DUS traits across three clusters. Clusters are color-coded as follows: I (blue), II (yellow), and III (green). Full names of the traits are provided in Table 1.


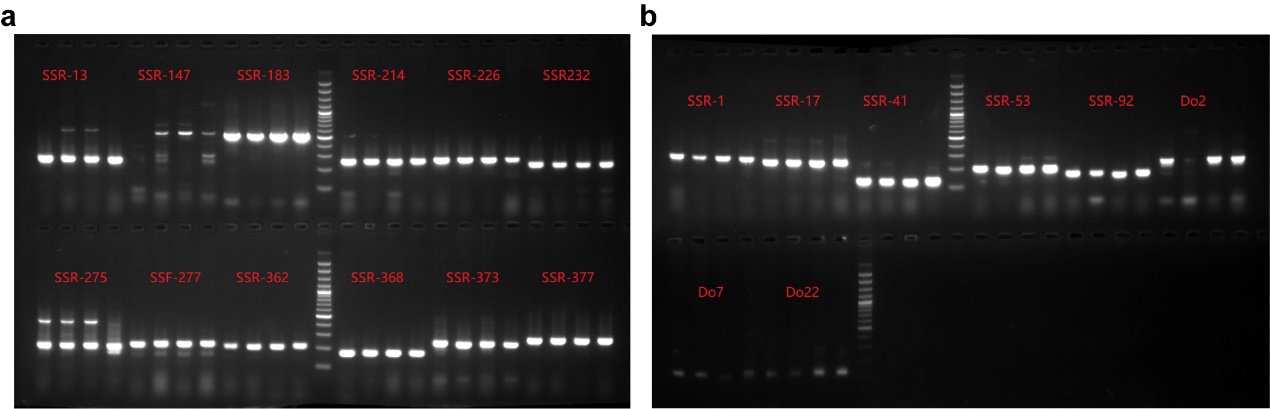


Figure S2. Gel electrophoresis images of amplified alleles from SSR-based molecular markers in random yam samples.


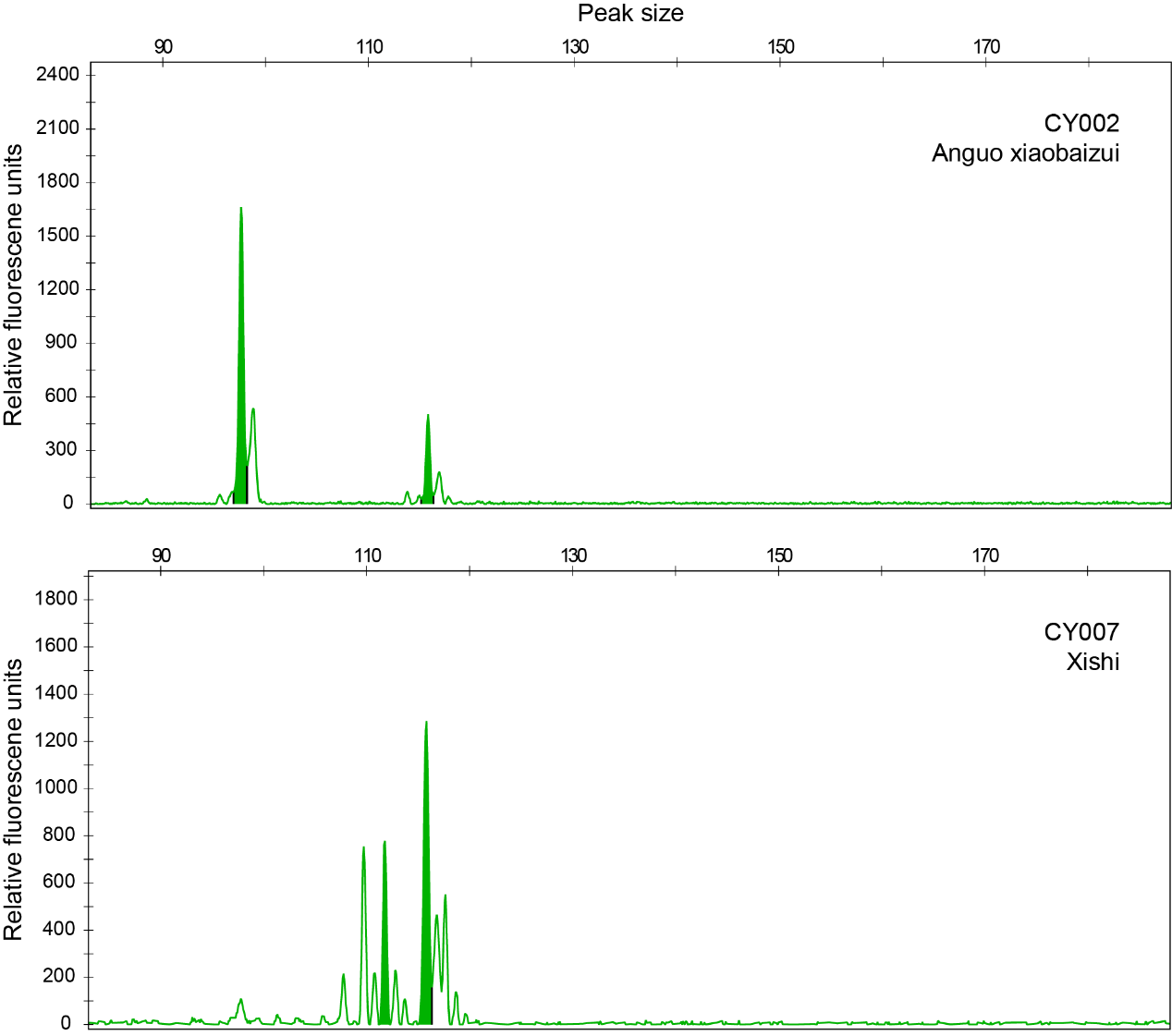


Figure S3. Electropherogram detected by Do7 for 2 yam accessions.


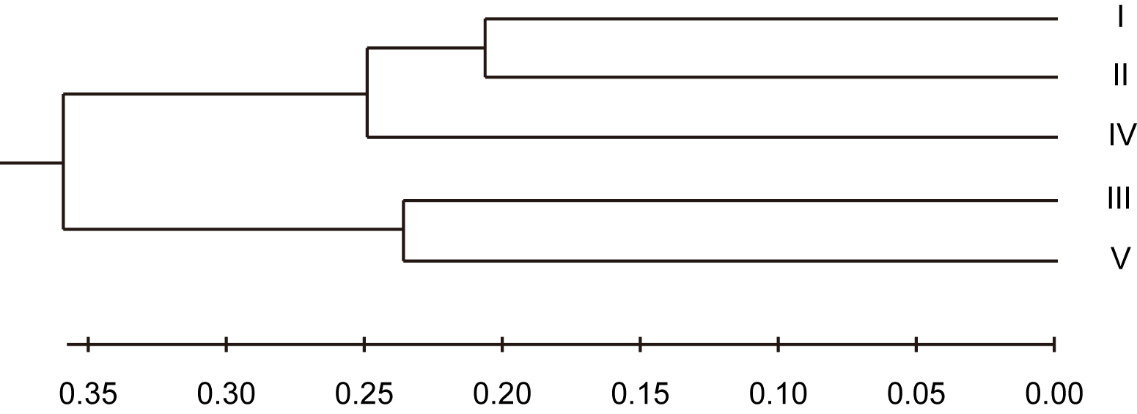


Figure S4. UPGMA phylogenetic tree illustrating genetic relationships among five groups, based on genetic distances derived from 19 SSR markers.


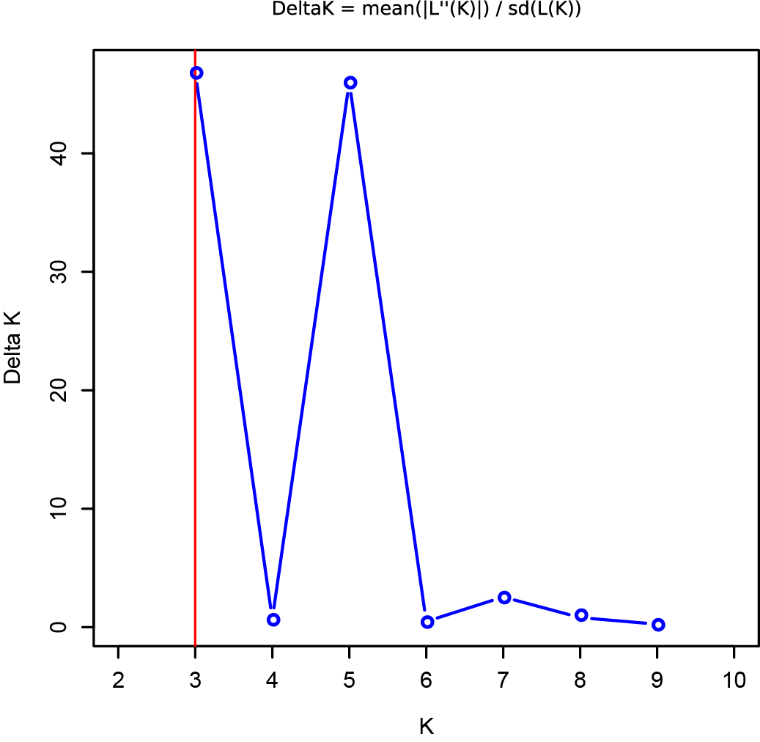


Figure S5. Genetic structure analysis in 113 yam accessions.
